# Polyphenolic Compounds in the Prevention and Treatment of Hypertension: A Review

**DOI:** 10.3390/ijms262110665

**Published:** 2025-11-01

**Authors:** Remigiusz Olędzki

**Affiliations:** Department of Biotechnology and Food Analysis, Wroclaw University of Economics and Business, Komandorska 118/120, 53-345 Wroclaw, Poland; remigiusz.oledzki@ue.wroc.pl

**Keywords:** hypertension, polyphenols, antioxidants, RAA system, angiotensin-converting enzyme

## Abstract

In recent years, there has been a growing consumer interest in natural food raw materials and the bioactive compounds they contain. Current research findings from leading research centres on the effects of polyphenolic bioactive compounds have sparked a discussion on the use of polyphenolic compounds as natural therapeutics in the prevention of lifestyle diseases, including cardiovascular diseases such as hypertension. This paper reviews original scientific research on the potential use of polyphenolic bioactive compounds in reducing primary and secondary hypertension. The paper also describes the mechanisms by which bioactive compounds act in the process of counteracting hypertension. Based on these observations, key therapeutic and dietary recommendations for the prevention of hypertension are formulated.

## 1. Introduction

Cardiovascular diseases (CVD), account for approximately one-third of all deaths worldwide [1]. These diseases are also responsible for almost one-third (32.4%) of all deaths in European Union countries [2]. Among the cardiovascular diseases that pose some of the most serious health problems and complications is hypertension.

Blood pressure is not a constant value and is subject to numerous fluctuations, for example, during aerobic exercise. At the same time, it is indicated that blood pressure values can often normalise in people with hypertension who regularly engage in physical activity [3]. Hypertension is defined as a condition when systolic blood pressure is above 140 mm Hg and/or diastolic blood pressure is above 90 mm Hg. Hypertension is diagnosed when systolic blood pressure (SBP) and diastolic blood pressure (DBP) values exceed the normal range and are confirmed by measurements taken at least two weeks after the initial consultation during which the abnormal blood pressure was observed. Hypertension gradually weakens the functioning of blood vessels, as mechanical stress, increased inflammation and oxidative stress lead to vascular endothelial dysfunction and damage to the arterial walls. As a result, atherosclerotic changes occur, reducing the elasticity of blood vessel walls. Hypertension also increases the production of collagen fibres and accelerates the degradation of elastin fibres in blood vessel walls. As a result, blood vessel walls lose their proper biomechanical properties, such as elasticity and plasticity. These changes secondarily activate the renin–angiotensin–aldosterone system (RAAS), resulting in a further increase in blood pressure [4].

Untreated hypertension can lead to pathological processes and changes in the body, such as heart failure, renal failure, retinopathy, lower limb ischaemia, stroke, coronary artery disease (ischaemic heart disease) and heart attack [5]. Thus, untreated patients with hypertension are at increased risk of death, mainly due to heart disease and cerebrovascular disease. People with very high blood pressure (>180/110 mmHg) may experience medical emergencies, such as collapse, which manifests itself in severe headaches and dizziness, shortness of breath and neurological disorders, which very often require rapid medical intervention [6]. One of the more serious complications is intracerebral haemorrhage, in which a blood vessel in the brain ruptures, causing bleeding into the brain parenchyma. This results in the formation of a haematoma, often with simultaneous leakage of blood into the ventricles of the brain [7]. People with long-term untreated hypertension are additionally at risk of acute aortic dissection, which can lead to serious complications such as aortic rupture (tearing of the aortic wall) [8]. Hypertension left untreated for many years (which may remain asymptomatic for a long time) can also lead to acute myocardial ischemia, left ventricular hypertrophy and dysfunction [9], and acute renal failure, which is often accompanied by oedema and the accumulation of serous fluid in the pleural cavity (so-called pulmonary congestion) [10].

Study results indicate that to prevent hypertension and its complications, regular blood pressure monitoring should be initiated, which allows for early detection of elevated blood pressure. The prevention and treatment of hypertension largely involves making recommended lifestyle changes, pharmacotherapy and nutrition. Based on a tabular meta-analysis of the results of 123 studies involving 613,815 participants, it has been proven that a 10-unit (10 mmHg) reduction in systolic blood pressure significantly reduces the risk of stroke by approximately 35% and heart failure by approximately 40% [11].

However, although lifestyle and dietary changes can have a very positive effect on overall health, they may not be sufficient to achieve normal blood pressure. It is therefore recommended to supplement the diet with bioactive compounds whose effectiveness in the prevention and treatment of hypertension has been experimentally confirmed.

The aim of this review article is the synthesis of the latest scientific reports (from the last 6 years) on the role of bioactive polyphenolic compounds found in natural plant materials in the prevention and supportive treatment of hypertension.

The scientific sources discussed in this article cover the most commonly studied groups of polyphenols, such as flavonoids, phenolic acids and stilbenes, which have been proven to have significant hypotensive effects. The selection of scientific reports discussed was largely based on the high reliability of the analysed scientific texts, which resulted from the analysed molecular and cellular mechanisms through which polyphenols modulate blood pressure, such as the effect on the endothelium of blood vessels, oxidative stress, the RAA system response, and changes in the elasticity and stiffness of arterial vessels.

Four scientific databases (Scopus, PubMed, Embase, Web of Science) were searched for articles on the effects of phenolic compounds from natural plant materials on hypertension. In order to narrow down the search for articles in which the assumed concept appears, the Boolean operator “AND” was used, by means of which the following combinations were entered into the search engines: “phenols AND arterial hypertension”, “phenolic AND arterial hypertension”, “polyphenols AND arterial hypertension” and “phenolic compounds AND arterial hypertension”. As a result, over 130 original scientific articles from 2019–2025 were analysed, from which data on the effect of phenolic compounds on primary and secondary hypertension were collected.

The obtained data were organised by plant material type and mechanism of vasodilation and then summarised in tables.

## 2. Polyphenolic Compounds—Characteristics and Presence in Food

**Figure 1:**
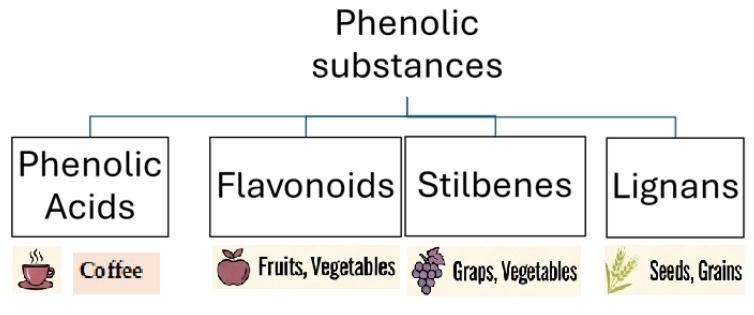
Four basic classes of phenolic compounds and plant raw materials that are their most common sources.

Clinical trials conducted worldwide indicate that various groups and forms of natural polyphenolic compounds may be effective in reducing hypertension of various aetiologies, i.e., caused by various primary and secondary factors [12]. There are four basic classes of phenolic compounds naturally occurring in plant raw materials: phenolic acids, flavonoids, stilbenes and lignans [13]. Figure 1 graphically presents four basic groups of phenolic compounds and the plant raw materials that are their most common sources [13].

### 2.1. Phenolic Acids

**Figure 2:**
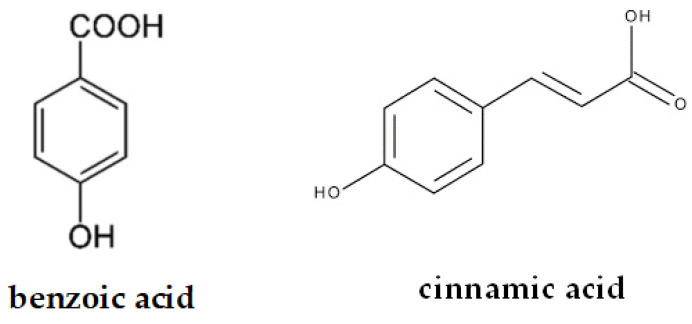
Phenolic acids comprise two subclasses, derivatives of benzoic acid and cinnamic acid.

Phenolic acids found in food are non-flavonoid phenolic compounds, divided into benzoic acid derivatives and cinnamic acid derivatives [14]. Benzoic acid derivatives naturally occur in raw materials such as chokeberry, blackcurrant, radish, grapes, strawberries, raspberries, onions, coffee fruit, and tea leaves [15]. Cinnamic acid derivatives, on the other hand, naturally occur in raw materials such as apples, plums, pears, potatoes, cabbage, lettuce, spinach, red wine, and olive oil [16]. Figure 2 graphically presents the two subclass groups of phenolic acids.

### 2.2. Flavonoids

**Figure 3:**
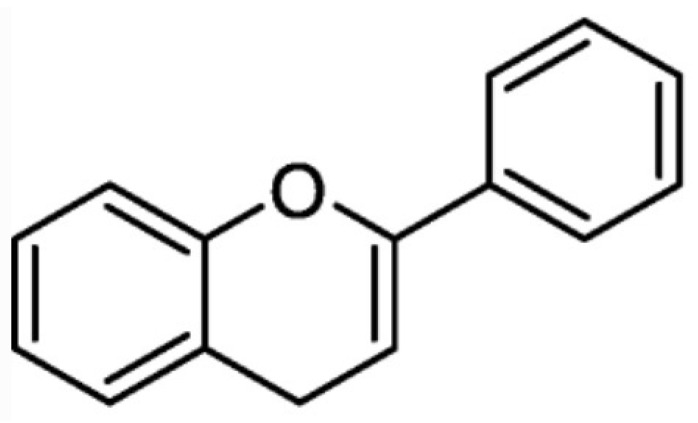
Basic structure of a flavonoid molecule.

Flavonoids are a group of polyphenolic compounds whose basic structure consists of two aromatic rings connected by three carbon atoms, which form an oxidised heterocyclic group—Figure 3 [17].

**Figure 4:**
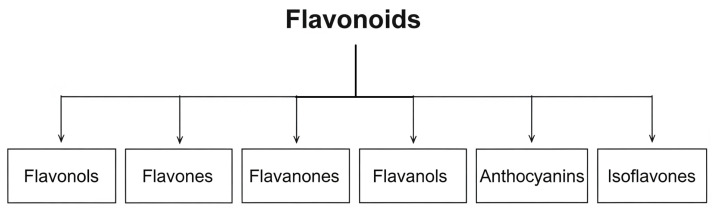
Main groups of flavonoids present in plant materials.

Due to their wide variety in structural composition, flavonoids are divided into six subclasses: flavonols, flavones, flavanones, flavanols, anthocyanins and isoflavones—Figure 4 [18,19,20].

The main types of flavonoids in the human diet are:

- Isoflavones: found in raw materials such as snap beans, green soybeans, bean sprouts and legumes [18,21].
- Flavonols: found in raw materials such as onions, apples, tea, lettuce, broccoli, dark grapes, elderberries, liquorice root, capers, cocoa beans and cabbage [19,20,21].
- Flavanols: found in raw materials such as green tea, dark chocolate (>70%), red wine, apples, barley and kiwi fruit [20,21,22].
- Flavones: found in raw materials such as celery, red pepper, parsley, lemon, pear and thyme [20,21,23].
- Flavanones: found in raw materials such as oranges, basil, dill weed, oregano, peppermint and grapefruit [20,21,24].
- Anthocyanins: found in raw materials such as grapes, cider vinegar, white wine, red wine, red onion, cherries, strawberries, blackcurrants, elderberries, chokeberry, berries of maqui and bilberry [20,21,25,26].

### 2.3. Lignans

**Figure 5:**
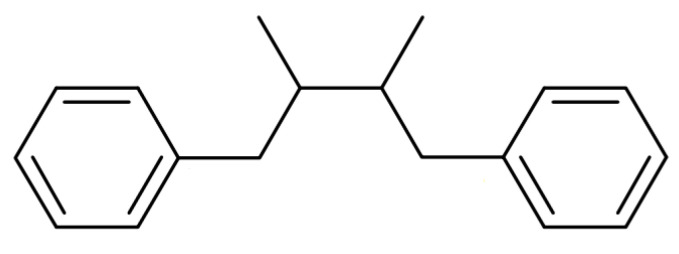
General structure of a lignan molecule.

Lignans are polyphenolic compounds found in large quantities in oilseeds. Rich sources include the seeds of common flax, Indian sesame, sunflower, and rapeseed [27,28]. They are also present in smaller amounts in cereals such as rice [29], as well as in some vegetables (e.g., asparagus) and fruits (e.g., grapes, kiwis, lemons, oranges, and pineapples) [30]. Chemically, lignans are diphenolic compounds derived from phenylpropanoids, which are synthesised in plants from shikimic acid (Figure 5) [31].

Some lignans, such as secoisolariciresinol and matairesinol, are considered phytoestrogens. It has been confirmed that secoisolariciresinol and matairesinol are converted by the microflora of the large intestine into enterolignans, such as enterolactone and enterodiol. Thus, secoisolariciresinol and matairesinol are precursor compounds for phytoestrogens, i.e., plant-derived chemical compounds which, due to their structure and functions, act similarly to female sex hormones—oestrogens [32].

### 2.4. Stilbenes

**Figure 6:**
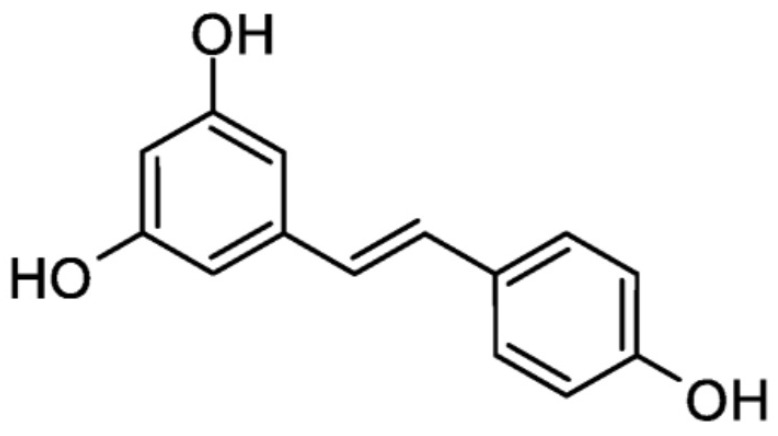
Basic structure of a stilbene molecule.

Stilbenes are polyphenolic compounds that contain two phenyl groups connected by a two-carbon methylene bridge—Figure 6 [33].

Stilbenes are present in plant materials due to their protective role against biotic (e.g., pathogens and herbivores) and abiotic (e.g., UV radiation and ozone) stress [34]. Most stilbenes act as phytoalexins, which are organic chemical compounds plants synthesise in response to biotic stress, particularly attacks by pathogens (viruses, bacteria, or fungi) [35]. The most studied naturally occurring stilbene is resveratrol (3,5,4′-trihydroxy-trans-stilbene), found mainly in grapes and red wine [36]. Resveratrol is also found in plant materials such as blueberries, cranberries, black mulberries, peanuts, and pistachios [37].

Table 1 presents phenolic compounds with antihypertensive activity and their source of origin.

## 3. Phenolic Compounds in the Control of Hypertension and Their Mechanisms of Action

### 3.1. Phenolic Compounds in the Treatment of Hypertension and Endothelial Dysfunction

It is estimated that the incidence of hypertension among adults identifying as Native Americans, Alaska Natives, multiracial, Black, and Hispanic in the Americas will increase from 51.2% in 2020 to 61.0% by 2050, affecting over 184 million adults in North and South America [66]. Therefore, natural ways of combating this condition are constantly being sought.

Recent scientific research suggests that certain polyphenol groups may be particularly effective. Epidemiological studies have long suggested a significant link between higher flavonoid intake and a lower risk of chronic diseases. A recent study (Australian Longitudinal Study of Women’s Health), which included two cohorts: a group of 6599 middle-aged women (average age of 52.5 years) and a group of 6099 women of childbearing age (average age of 27.5 years), confirmed that increased consumption of flavones, isoflavones, and flavanones reduces the incidence of hypertension in the middle-aged women. In the younger women, however, higher consumption of flavanols is key to reducing the incidence of hypertension [41]. The main dietary sources of these beneficial flavonoids were oranges, orange juice, apples, red wine, and soya milk, highlighting the importance of appropriately selected nutritional intervention in improving cardiovascular health in women [67].

Studies conducted on a large US population (15,752 participants aged 20–60; 7408 with hypertension) confirmed that an increased total flavonoid intake (specifically flavan-3-ols, anthocyanidins, and flavonols) of 235.67 mg/day significantly reduces the risk of developing or having hypertension compared to a low-intake group. This increased intake was particularly effective in reducing hypertension incidence among people without comorbidities and those under the age of 60. Furthermore, in participants without hypertension (8344 total), an increase in total flavonoid intake resulted in a reduction in C-reactive protein (hsCRP), a main inflammation biomarker. However, this increase in total flavonoid intake did not cause changes in other metabolic risk factors, such as HbA1c, fasting glucose, triglycerides, total cholesterol, or LDL/HDL fractions [68].

Studies on animal models show that both primary flavonoids and their metabolites exhibit antihypertensive activity. One such metabolite, 3-hydroxyphenylacetic acid (3-HPAA), formed by the conversion of dietary flavonoids (specifically quercetin) by the gut microbiota, may be effective in treating essential hypertension [69]. It has been confirmed that 3-HPAA, formed directly from 3,4-dihydroxyphenylacetic acid (DHPA), has a strong vasodilatory effect on blood vessels ex vivo without significantly affecting heart rate. This effect was dose-dependent and decreased both the mean systolic and mean diastolic blood pressure in test animals (rats). The mechanism of this vasodilatory action, including on the coronary arteries, is associated with increased release of nitric oxide (NO) by the vascular endothelium, resulting from the activation of endothelial Nitric Oxide Synthase (eNOS) [69].

**Figure 7:**
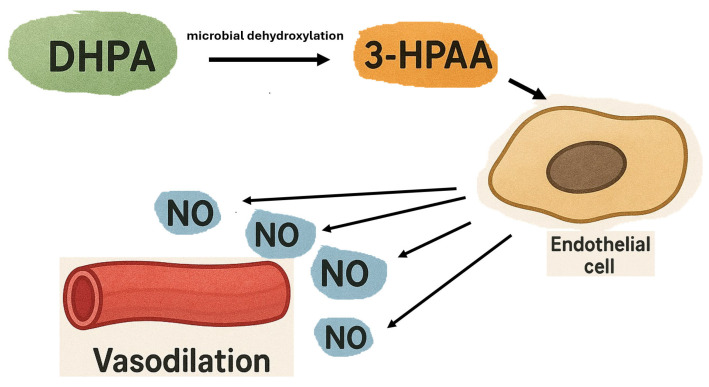
Mechanism of the vasodilatory action of 3-hydroxyphenylacetic acid (3-HPAA) on arterial vessels. DHPA—3,4-dihydroxyphenylacetic acid, 3-HPAA—3-hydroxyphenylacetic acid, NO—nitric oxide [69].

Figure 7 graphically presents the mechanism of the vasodilatory action of 3-hydroxyphenylacetic acid (3-HPAA) on arterial vessels.

Despite its vasodilatory effect, 3-hydroxyphenylacetic acid (3-HPAA) showed no effect on cyclooxygenase or L-type calcium channels [70], which are the main route of Ca^2+^ influx causing vascular muscle contraction [71]. Since dietary flavonoids are poorly absorbed in the small intestine, bacterial biotransformation in the colon can significantly increase their bioavailability [72]. Other quercetin metabolites formed in the large intestine, such as 3,4-dihydroxyphenylacetic acid (DHPA) and 4-methylcatechol (4MC), may also have blood pressure-lowering effects in essential hypertension. Simultaneous intravenous infusions of 4-methylcatechol (4MC) and 3-(3-hydroxyphenyl)propionic acid (3HPPA) in spontaneously hypertensive rats produced a marked and long-lasting blood pressure reduction. However, the simultaneous use of 3HPPA and DHPA also caused a significant blood pressure reduction, but the effect was short-lived (approx 30 s) [73]. The mechanism of action for 4-methylcatechol (4MC) is independent of nitric oxide (NO). It involves a reduction in the intracellular Ca^2+^ concentration (by closing calcium channels and inhibiting Ca^2+^ influx) and the activation of K^+^ channels, leading to K^+^ efflux and subsequent vasodilation [74]. This phenomenon reduces peripheral vascular resistance, decreasing blood pressure [75].

It has been confirmed that polyphenols present in plants such as roselle (*Hibiscus sabdariffa*) and lemon verbena (*Lippia citriodora*) can also significantly counteract high blood pressure. The results of a study of 80 people who consumed phenolic extract from roselle flowers (*Hibiscus*) and *Lippia trifoliata* for 84 days showed that phenolic compounds effectively reduce primary hypertension, even among people with a sedentary lifestyle [76].

The calyxes of roselle and the leaves of lemon verbena have been shown to be rich in anthocyanins and phenylpropanoids, such as verbascoside (a phenylpropanoid glycoside) and isoverbascoside (a phenylethanoid glycoside), which have strong antihypertensive and anti-inflammatory properties. Among the antihypertensive compounds, large amounts of anthocyanins, such as delphinidin-3-O-sambubioside and cyanidin-3-O-sambubioside, have also been isolated. It has been observed that daily consumption of phenolic extract containing 500 mg of polyphenols (175 mg of polyphenols from *Hibiscus sabdariffa* flowers and 325 mg of polyphenols from *Lippia trifoliata* leaves) significantly reduces mean blood pressure, especially systolic blood pressure (SBP), by an average of 3.76 mm Hg. The observed synergistic effect in lowering systolic blood pressure is explained by the ability of the polyphenols contained in *Hibiscus sabdariffa* and *Lippia citriodora* (mainly catechins and epicatechins) to increase the activity of endothelial nitric oxide synthase (eNOS), resulting in increased vasodilation. In addition, polyphenols contained in *Hibiscus sabdariffa* and *Lippia citriodora* may inhibit the activity of angiotensin-converting enzyme I, thereby reducing the activity of angiotensin II. Furthermore, it has been suggested that polyphenols derived from *Hibiscus sabdariffa* and *Lippia citriodora* may increase the expression of the adiponectin and PPAR-α genes while simultaneously reducing the level of NF-kB (nuclear factor kappa B) protein. Adiponectin is a powerful anti-inflammatory factor that inhibits the activation of inflammatory pathways involving NF-kB and thus reduces the production of pro-inflammatory cytokines. In turn, PPAR-alpha (peroxisome proliferator-activated receptor alpha) is a protein transcription factor that negatively regulates pro-inflammatory signalling pathways (such as AP-1 and NF kappa B) and thus inhibits the expression of pro-inflammatory cytokines, e.g., IL-6, which also protects the blood vessel endothelium from inflammation [76].

Research results indicate that polyphenolic compounds from the Risso & Poiteau variety bergamot (*Citrus bergamia*), grown in Calabria, Italy, can effectively reduce hypertension, even with high salt intake [51]. Experiments on an animal model (spontaneously hypertensive rats susceptible to stroke, SHRSP) showed that bergamot polyphenols reduce damage to primary cerebral endothelial cells and eliminate oxidative stress caused by high NaCl concentrations. These compounds also improve the viability of endothelial cells and increase the formation of new, small blood vessels within the brain. Furthermore, these compounds can accelerate the healing of wounds in brain vessels by stimulating the migration of cerebral endothelial cells [51]. Bergamot polyphenols also help maintain the normal mitochondrial membrane potential (approx 150 mV) in endothelial cells, which is essential for ATP synthesis. The protective effect is suggested to be due to flavonoid glycosides such as neoeriocitrin, neohesperidin, and naringin, and glycosylated polyphenols such as bruteridin and melitidin [51].

It has been shown that phenolic compounds contained in the fruit of the colocynth (*Citrullus colocynthis*) may also have an antihypertensive effect. Studies using an animal model (SHR rats) have shown that consumption of *Citrullus colocynthis* fruit may have an antihypertensive effect due to the high content of flavonoids, especially flavones, in this raw material. Analysis of phenolic extracts obtained from *Citrullus colocynthis* fruit (LCMS) revealed the presence of numerous phenolic compounds, such as gallic acid, p-hydroxybenzoic acid, p-coumaroylquinic acid, chlorogenic acid, caffeic acid, vanillic acid, syringic acid, sinapic acid and ferulic acid. In addition, compounds such as epicatechin, hesperidin, resveratrol, rutin, isoquercetin, kaempferol-3-glucoside, myricetin-3-O glucuronide, myricetin-3-O-pentoside, quercetin-3-O-glucuronide, eriodictyol-7-O-rutinoside and apigenin glucoside [46]. It has been confirmed that polyphenolic compounds present in *Citrullus colocynthis* fruit can significantly reduce mean arterial pressure, systolic blood pressure (SBP), diastolic blood pressure (DBP) and pulse pressure. In animal model studies, it was also observed that the intake of phenolic compounds at a dose of 500 mg per kg of body weight causes a significant improvement in the electrocardiogram (ECG) pattern, and an improvement in pulse wave velocity (PWV) to values in the range of 5 to 15 m/s [46]. Failure to maintain these two parameters (ECG and PWV) is considered a significant indicator of cardiovascular risk [77].

It has been confirmed that polyphenolic extracts from the leafy stem of *Flemingia fagina*, a medicinal leguminous plant commonly used in traditional medicine in many West African countries, may be effective in treating hypertension [43].

Ex vivo studies conducted on an animal model (male and female mice) showed that polyphenolic extracts from the leafy stem *F*. *faginea* of have a strong vasodilatory effect, including on the thoracic aorta. It has been shown that polyphenolic compounds from *F. fagina* induce long-lasting relaxation of blood vessels by inhibiting the influx of Ca^2+^ ions through the cell membrane, which contributes to a decrease in the intracellular concentration of Ca^2+^. It has been confirmed that polyphenolic compounds from *F. fagina* inhibit the release of Ca^2+^ stored in the sarcoplasmic reticulum by inhibiting the activity of specific G protein-coupled receptors at the level of vascular smooth muscle cells [43].

It has also been suggested that polyphenolic compounds from *F. fagina* may have a vasodilatory effect not only at the level of vascular smooth muscle cells (by reducing Ca^2+^ concentration), but may also interact with the endothelium in the release of chemical mediators of vascular relaxation, such as nitric oxide (NO), prostacyclin (PGI_2) and EDHF (Endothelium-Derived Hyperpolarising Factor) [43]. PGI_2 has the ability to bind to PGI_2 receptors on the surface of smooth muscle cells, leading to their relaxation. In turn, the EDHF factor binds to receptors on the surface of vascular smooth muscle cells, leading to the opening of K^+^ channels. The efflux of K^+^ ions leads to hyperpolarisation of the cell membrane. This hinders the activation of calcium channels and leads to a decrease in the concentration of Ca^2+^ inside the smooth muscle cell. As a result, the smooth muscles of the vessel walls relax and the blood vessels dilate [78].

**Figure 8:**
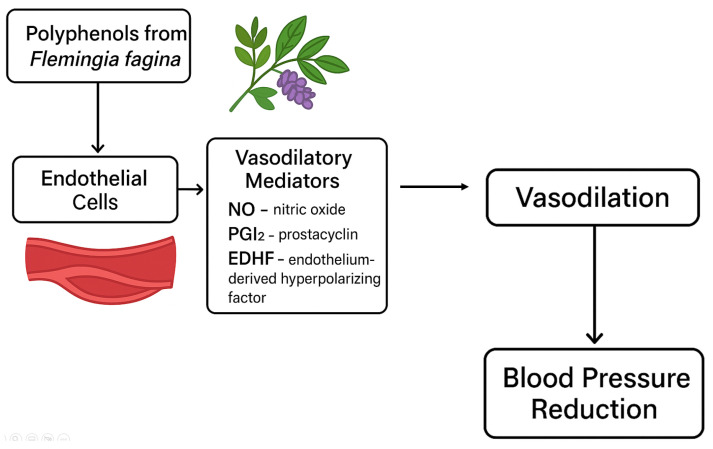
Mechanism of vasodilatory action of phenolic compounds derived from the leafy stem of *Flemingia faginea* Guill. & Perr. through the process of release of chemical mediators of vascular relaxation from the endothelium [43].

Figure 8 graphically presents the mechanism of vasodilatory action of phenolic compounds derived from the leafy stem of *Flemingia faginea* Guill. & Perr [43].

The results of phytochemical studies have shown that the therapeutic effect described above is due to the polyphenolic compounds present in the leafy stems of *Flemingia fagina*, mainly flavonoids and phenolic acids, such as myricetin rhamnoside, myricetin rutinose, quercetin rutinose, caffeoyl glucoside, 5-caffeoylquinic acid and gallic acid glycoside. In addition to their vasodilatory properties, these phenolic compounds are also characterised by a high ability to scavenge oxygen free radicals formed within the endothelium of blood vessels [43].

It has been confirmed that polyphenolic compounds contained in barberry berries (*Berberis vulgaris L.*) can significantly reduce the risk of organ and tissue complications resulting from hypertension. Based on a randomised, controlled study (involving 84 hypertensive people; average age 54, BMI 28 kg/m, it was shown that consuming 10 g of dried barberry per day improves blood vessel endothelial function and reduces inflammatory processes in arterial vessels. Consuming barberry reduces (by 52.2%) the plasma concentration of the pro-inflammatory mediator macrophage/monocyte chemotactic protein-1 (MCP-1) and reduces (by 58.76%) the concentration of the adhesion molecules vascular cell adhesion molecule-1 (VCAM-1) and intercellular adhesion molecule-1 (ICAM-1) in blood vessels. It has been confirmed that the concentration of VCAM-1 and ICAM-1 increases in response to inflammation, especially in areas where blood flow is impaired, e.g., in atherosclerosis [64]. It is suggested that the beneficial effects of barberry fruit are closely related to its high content of polyphenolic compounds (11.96 mg gallic acid equivalent per gram of dried product). It is indicated that the observed antihypertensive and anti-inflammatory effects are due to the flavonoids (rutin and quercetin), anthocyanins (cyanidin-3,5-diglucoside, cyanidin-3-glucoside, delphinidin-3,5-diglucoside, petunidin-3-O-β-D-glucoside, pelargonidin-3,5-diglucoside and pelargonidin-3-glucoside), and phenolic acids (ellagic acid, chlorogenic acid and caffeic acid) [64,65]. It has also been determined that a beneficial therapeutic effect can be achieved with a dose of 10 g of dried barberry powder consumed daily for 60 days [64].

It has been confirmed that wine lees (wine sediment), a by-product of wine production, can inhibit angiotensin-converting enzyme (ACEi) and, as a result, lower blood pressure. Studies in animal models (spontaneously hypertensive Wistar-Kyoto rats) have shown that phenolic wine lees from Cabernet grape wine production can reduce ACEi activity by up to 56%. Lees from Garnacha and Mazuela varieties also showed high ACEi reduction capacity (44% and 50%, respectively). It is suggested that this effect is due to polyphenolic compounds such as catechin, epicatechin, procyanidin dimers B2 and iso1, whose presence was confirmed in the supernatant from Cabernet wine lees. The presence of quercetin, isorhamnetin, gallic acid, trans-resveratrol, and piceatannol was also confirmed, along with anthocyanins, primarily malvidin-3-glucoside and its acetyl and coumaroyl derivatives [60]. It was observed that after oral administration of Cabernet wine lees supernatant (at a dose of 5 mL/kg body weight), there was a significant reduction in blood pressure in animals, with a therapeutic effect comparable to the drug Captopril. Furthermore, the hypotensive effect may persist for 24 to 48 h. At the same time, no hypotensive effect was observed in normotensive Wistar-Kyoto rats, which confirms the safety of these polyphenols. It has been established that a daily amount of 73 mL of phenolic extract (from Cabernet wine lees) consumed by a person weighing 70 kg should also effectively lower blood pressure in cases of diagnosed hypertension [60].

It has been confirmed that phenolic compounds extracted from *Alchemilla viridiflora*, a herbaceous plant widespread in Central Greece, Bulgaria, North Macedonia, and Serbia, may also be effective in treating hypertension. It has been shown that phenolic compounds contained in A. *viridiflora* extract can inhibit angiotensin-converting enzyme I (ACE I). This is because compounds such as tiliroside, ellagitannins, ellagic acid pentoses, galloyl-hexahydroxydiphenyl-glucose, and myquellianin (quercetin 3-O-β-D-glucuronide) have a strong affinity for the active site within the ACE I molecule, causing enzyme inhibition [49].

It has been shown that consumption of numerous varieties of turnip (*Brassica rapa*) may also reduce the risk of hypertension by inhibiting angiotensin-converting enzyme I (ACE I) activity. GC-MS and in silico analysis of *Brassica rapa* leaves have shown that the antihypertensive potential is due to the presence of polyphenols, particularly flavonoids (at a concentration of 259.13 mg/g of dry leaves). It has been confirmed that the phenolic substance in *Brassica rapa* leaves that exhibits strong antihypertensive properties is primarily 4-ethynyl-2,6-dimethoxyphenol, a polyphenol that is a product of ferulic acid metabolism, which can inhibit the activity of ACE I [41].

It has been proven that the fruits of the highbush blueberry (*Vaccinium corymbosum*, Tifblue variety) and the wild lowbush blueberry (*Vaccinium virgatum*, Rubel variety) also have antihypertensive effects, due to their high polyphenol content and antioxidant capacity [54]. It is suggested that the antihypertensive mechanism is related to the attenuation of angiotensin II-induced phosphorylation of MAPKs (Mitogen-Activated Kinases), mainly SAPK/JNK and p38. These kinases are stimulated by oxidative stress and play a role in atherosclerosis pathogenesis by mediating inflammation, endothelial activation, and smooth muscle cell proliferation [61,62]. Polyphenolic compounds from both blueberry species have the ability to weaken the phosphorylation of kinases responsible for the production of EDCF (Endothelium-Derived Contracting Factors) [54]. In addition, phenolic compounds from both species increase the expression of the NRF2 transcription factor, which activates the expression of antioxidant response genes like SOD1 and NQO1 [54,79]. Phenolic compounds also increase the expression of heme oxygenase-1 (HO-1), which has anti-inflammatory effects and protects against vascular remodelling. It has also been shown that polyphenolic compounds in blueberries can weaken the angiotensin II-induced phosphorylation of the NF-κB p65 protein, reducing the expression of pro-inflammatory genes [54].

As a result, phenolic compounds from both blueberry species lead to a reduction in oxidative stress and an increase in nitric oxide (NO) levels in blood vessel endothelial cells through an NRF2-dependent mechanism [54]. It has been confirmed that the main polyphenolic compounds responsible for the antihypertensive effect are anthocyanins, such as cyanidin-3-O arabinoside, cyanidin-3-O-galactoside, malvidin-3-O-arabinoside, mal-vidin-3 O-galactoside, malvidin-3-O-glucoside, paeonidin-3-O-galactoside, paeonidin 3-O-glucoside and petunidin-3-O-galactoside [54,55].

It is indicated that phenolic compounds contained in fermented flour from Tuscan *Triticum dicoccum* wheat, such as gallic acid, quercetin-3 rutoside (rutin), transferolic acid, isoquercitrin and quercetin may be directly responsible for inhibiting the conversion of angiotensin I to active angiotensin II. Additionally, an experiment using a human colon cancer cell line (HT-29) showed that bioactive polyphenols from fermented Tuscan wheat flour have a protective anti-inflammatory effect by reducing the expression of the inflammatory mediator IL-8 (Interleukin 8) [62]. It has been confirmed that if IL-8 is produced in excessive amounts, it can activate endothelial cells, leading to increased expression of adhesive molecules (surface glycoproteins) such as ICAM-1 and VCAM-1, which facilitate the adhesion of immune system cells (e.g., leukocytes) to the blood vessel wall [80].

Research indicates that polyphenolic compounds contained in the leaves, flowers and fruits used in West African traditional medicine of plants such as Carissa edulis Vahl (commonly known in the African language isiZulu as ‘umgabunkhomo’ or ‘mutsambatsi’), Diodia scandens Sw. (locally known as the ‘akan-nsisiri’ plant) and Cleome gynandra L. (locally known as the ‘African spider flower’) may exhibit significant biological activity in the cardiovascular system [56].

As a result of studies conducted on animal models (rats and pigs), it has been confirmed that polyphenolic extracts from the leaves of *C. edulis*, *D. scandens* and *C. gynandra* can improve the functioning of the main blood vessels in the body [66]. It is indicated that polyphenols contained in the described plants can strongly induce the relaxation of the left coronary artery and the main artery (aorta) as a result of stimulating endothelial nitric oxide synthase (eNOS) to produce NO in the walls of these arteries. It is suggested that the relaxation of blood vessels is caused by phenolic compounds present in the above-ground parts of *C. edulis*, *D. scandens* and *C. gynandra*, such as patuletin 7-(6″-(2-methylbutyryl)-glucoside), catechin 3-O-rutinoside and 6-C glucosylquercetin. These compounds may accumulate preferentially in the endothelium of the coronary artery and aorta walls and induce vasodilation mediated by the endothelial pathway ‘nitric oxide synthase-NO-guanylate cyclase-protein kinase G, the activation of which leads to the relaxation of the smooth muscles of the blood vessel walls [56].

It has been observed that polyphenolic compounds contained in the leaves of *Ziziphora clinopodioides subsp. bungeana* (Juz.) Rech.f., a widespread subshrub growing in north-eastern China, Kazakhstan, Kyrgyzstan, Mongolia, Russia, Tajikistan, Turkmenistan and Uzbekistan, may be highly effective in counteracting diseases such as coronary heart disease and hypertension. The results of studies conducted on animal models indicate that the phenolic compounds present in the leaves, such as pinocembrin-7-O-rutinoside, chrysin-7-O-rutinoside, acacetin-7-O-rutinoside, luteolin-7-O-rutoside, rutin, rosmarinic acid and cinnamic acid derivatives may be responsible for the antihypertensive activity. Polyphenols present in the extract may dilate blood vessels by stimulating bradykinin B2 receptors on the surface of endothelial cells and by activating the NO synthase-NO synthesis-guanylate cyclase-protein kinase G pathway [57].

It has also been shown that a polyphenolic extract from the aboveground parts of *Anvillea radiata*, a plant endemic to Morocco and Algeria, can significantly lower blood pressure. In an animal model study, it was confirmed that the extract significantly reduced systolic blood pressure (SBP), mean blood pressure (MBP), and diastolic blood pressure (DBP), while having no significant effect on heart rate (HR). The extract was confirmed to have vasorelaxant effects in isolated rat aortic rings [81]. It has been suggested that compounds such as chlorogenic acid and caffeic acid contained in this plant are responsible for the observed antihypertensive effect. Chlorogenic acid and caffeic acid are indicated to have a vasorelaxant effect on blood vessels by closing calcium channels and reducing the influx of calcium ions into the vascular smooth muscle cells. A second suggested mechanism for the vasorelaxant action is the direct activation of the nitric oxide (NO) synthesis pathway through stimulation of endothelial nitric oxide synthase (eNOS). Consequently, the key signalling mechanism, the NO/cGMP pathway, is activated, where NO activates soluble guanylyl cyclase (sGC), converting GTP to cGMP. An increase in intracellular cGMP concentration leads to the activation of protein kinase G (PKG), which causes vascular smooth muscle relaxation. Consequently, the activation of the NO/cGMP pathway by polyphenolic compounds in *A. radiata* may be part of the prevention and treatment of hypertension [81].

It has been shown that lentisk pistachio (*Pistacia lentiscus L.*), traditionally used for its diuretic properties, has also been shown to be effective in the treatment of hypertension. Analysis of the essential oil from the leaves showed that the main polyphenolic constituents are 3,5-di-O-galloylquinic acid, gallic acid and 3,4,5-tri-O-galloylquinic acid. It has been confirmed that these compounds can inhibit the activity of NADPH oxidase, a major source of reactive oxygen species (ROS), which are central to the pathogenesis of hypertension. The production of large amounts of ROS (such as superoxide anion radical (O_2_^-∙^) or hydrogen peroxide (H_2_O_2_) significantly interferes with vascular wall smooth muscle cell function. Therefore, inhibition of NADPH oxidase by these polyphenols is an important element in counteracting chronic inflammation, which promotes hypertension [82]. It has been shown that these phenolic acids have a high absorption capacity in the gastrointestinal tract and a high level of passive diffusion across the blood–brain barrier (BBB). This means these compounds can easily diffuse into the central nervous system vessels, which determines their high therapeutic potential [38].

It has also been confirmed that *Lumnitzera racemosa*, commonly known as black mangrove, a plant cultivated in south-east Africa, the western Indian Ocean, and western Pacific countries, may also be effective in the treatment of hypertension [40]. It has been suggested that polyphenolic compounds such as myricetin and rosmarinic acid, which exhibit potent antioxidant and anti-inflammatory properties, are responsible for the antihypertensive activity and are present in abundance in the leaves of *L. racemosa* [40].

Studies conducted on an animal model (male Swiss albino mice) suggest that also 3-hydroxyflavone (flavone-3-ol), found in abundance in the spotted orchid (*Dactylorhiza maculata*), may exhibit antihypertensive effects. This substance may have a high therapeutic potential due to its strong antioxidant properties, resulting from the presence of a 3-hydroxy group within the heterocyclic C-ring. It has been indicated that 3-hydroxyflavone may have a protective function towards the vascular endothelium largely through properties related to the neutralisation of free oxygen radicals (ROS), an excess of which leads to endothelial dysfunction and, consequently, activation of the renin–angiotensin–aldosterone (RAA) system [83].

It has been shown that phenolic compounds from the skins and seeds of muscatel grapes can prevent hypertension-induced heart damage and offset the accompanying oxidative stress. It has been confirmed that polyphenols from muscatel grape skins and seeds can enhance left ventricular diastolic function by lowering filling pressures. In an animal model study, it was shown that these polyphenols can prevent cardiac damage by limiting the increase in mean cross-sectional area of cardiomyocytes that accompanies hypertension. The compounds primarily responsible are epicatechin, gallic acid, procyanidin B, ellagic acid, catechin, and catechin gallate. Supplementation with the extract reduces the amount of 4-hydroxynonenal and malondialdehyde in cardiomyocytes, both formed by lipid peroxidation. In addition, consumption of the extract has been confirmed to increase the activity of superoxide dismutase (SOD-1), protecting cardiomyocyte mitochondria under oxidative stress. Consumption can also cause a significant increase in catalase (CAT) activity in heart tissue as a result of an almost 2-fold increase in catalase mRNA expression. It has been suggested that an effective total dose of polyphenols that can cause a cardioprotective effect in humans weighing about 60 kg is about 2.3 mg/kg body weight per day [48].

It has been observed that the combined intake of polyphenols and other dietary components such as L-citrulline can enhance the beneficial vasodilatory effect and thus reduce the risk of developing hypertension. Based on results from a double-blind, randomised clinical trial, even short-term (six weeks) combined consumption of a polyphenol extract extracted from cranberries (*Vaccinium macrocarpon*) and grape seeds (*Vitis vinifera*) at 548 mg/day combined with L-citrulline at 2 g/day was shown to significantly reduce systolic blood pressure (SBP) and cardiovascular disease markers. The observed SBP reduction was related to both daily and 24 h blood pressure values in adult women with a clinically confirmed prehypertensive state [84].

L-citrulline, a naturally occurring amino acid, stimulates nitric oxide (NO) production by increasing L-arginine production [85]. The findings suggest that women’s bodies may respond more sensitively to the combined effects of polyphenols and L-citrulline than men’s bodies. This may be related to significantly higher amounts of oestrogen, which hormones have been shown to have antihypertensive properties. Oestrogen induces vasodilation and lowers blood pressure by promoting the activation of endothelial nitric oxide synthase and by reducing the number of type 1 receptors for angiotensin II and receptors for endothelin 1 (ET1) [86].

### 3.2. Polyphenols in the Reduction in Hypertension Associated with Pregnancy Complications

It has been shown that trans-resveratrol, extracted from grape skins, can significantly reduce the symptoms of pre-eclampsia, a multisystem disease in pregnant women characterised by hypertension after 20 weeks of pregnancy. It is indicated that the therapeutic effect of resveratrol in regulating blood pressure in pre-eclampsia is related to modulation of cytokines and inhibition of oxidative stress and inflammation within the blood vessels [87]. Studies have shown that resveratrol increases the expression of vascular endothelial growth factor (VEGF) in the placenta [87], which stimulates angiogenesis leading to lower blood pressure [88]. At the same time, resveratrol inhibits the release of the soluble receptor sFlt1, which is an anti-angiogenic factor and acts as an antagonist against VEGF [88].

Clinical studies have shown that resveratrol is both a safe and effective adjunct (adjuvant) to orally administered nifedipine, a drug used to relieve hypertensive symptoms in women with pregnancy-induced hypertension. A meta-analysis showed that combination treatment with resveratrol and nifedipine can reduce the treatment time required to achieve normal blood pressure (compared to using nifedipine alone) [88]. In addition, the concomitant use of resveratrol and nifedipine allows the use of a lower dose of nifedipine to demonstrate therapeutic efficacy [89].

Preeclampsia is also associated with increased activation of the vascular endothelium and, as a result, the release of inflammatory factors (TNF-α, IL-6, IL-1β) [90]. Resveratrol has also been confirmed to reduce the activity of TNF-α (tumour necrosis factor alpha), a pro-inflammatory cytokine. Reduced TNF-α activity at the cellular level, in turn, leads to reduced synthesis of cytokines, especially IL-1 and IL-6, which are crucial to the inflammatory process. Their reduced concentrations are indicative of a decreasing severity of inflammatory diseases. IL-1 and IL-6 can affect endothelial cell dysfunction, which involves impaired nitric oxide (NO) production, leading to increased vascular resistance and elevated blood pressure [88]. In addition, IL-1 and IL-6 stimulate the activity of the renin-angiotensin system, where increased production of angiotensin II consequently leads to vasoconstriction and increased blood pressure [91].

At the same time, it has been observed that resveratrol decreases the activity of STAT (Signal Transducer and Activator of Transcription) proteins. STAT proteins have been confirmed to be involved in the regulation of inflammatory responses and the formation of chronic inflammatory conditions, which consequently contribute to the development of hypertension. In addition, STAT proteins can alter the structure of blood vessels, thereby leading to increased vascular resistance. It has been confirmed that resveratrol can also inhibit overactivation of the EGFR receptor, an epithelial growth factor receptor. Excessive activation of EGFR leads to proliferation of smooth muscle cells in blood vessels, which can lead to narrowing of the vascular lumen and increased vascular resistance [87].

Studies indicate that the efficacy of resveratrol can be significantly enhanced by esterifying it with butyric acid to form resveratrol butyrate ester. It has been confirmed that resveratrol butyrate has higher bioavailability and higher antioxidant activity than unmodified resveratrol [92].

Resveratrol butyrate may be effective in reducing hypertension that occurs early in foetal life due to maternal exposure to toxic chemicals [92]. In animal model studies, resveratrol butyrate was confirmed to be effective in protecting offspring from this hypertension by reducing oxidative damage to the kidney and by increasing the expression of the short-chain fatty acid (SCFA) receptor [78]. SCFAs, produced by intestinal bacteria, activate FFAR3 (GPR41) receptors found in vascular smooth muscle cells, resulting in vasodilation and a reduction in blood pressure [92,93].

It has been indicated that resveratrol may play an important role in reducing hypertension in individuals born after intrauterine growth restriction (IUGR). Animal studies confirmed that IUGR syndrome causes severe complications in the form of oxidative stress, premature ageing, and hypertension [94].

It has been confirmed that, in endothelial colony-forming cells (ECFCs) from organisms with or after IUGR, resveratrol increases proliferation and improves capillary blood vessel formation. Furthermore, resveratrol stimulates nitric oxide (NO) production by increasing the expression of eNOS genes [94]. It has also been observed that resveratrol increases the expression of Cu/Zn superoxide dismutase (SOD), indicating that it can significantly increase NO production by preventing free radical damage to eNOS [94,95].

### 3.3. Polyphenols in the Treatment of Hypertension During Metabolic Syndrome

The results of a study on a group of 143 people with confirmed metabolic syndrome showed that phenolic compounds contained in the fruit of chokeberry (*Aronia melanocarpa*) may be effective in the prevention and treatment of hypertension. It was confirmed that a 4-week supplementation with a standardised fruit extract of *Aronia melanocarpa* L. at a dose of 400 mg of polyphenols per day could significantly reduce both systolic and diastolic blood pressure. In addition, it has been observed that consumption of phenolic compounds contained in chokeberry fruit for 2 weeks can significantly reduce serum total cholesterol levels in women with confirmed type 2 diabetes. Furthermore, the consumption of chokeberry fruit polyphenols by women with confirmed metabolic syndrome and type 2 diabetes has been observed to significantly reduce low-density lipoprotein (LDL) and blood triglyceride levels [96].

It is indicated that the antihypertensive and metabolic syndrome-preventing effects of chokeberry fruit are due to the phenolic compounds, such as phenolic acids, proanthocyanidins, anthocyanins, flavonols and flavanones, contained in this raw material (in a total amount of up to 2466 mg per 100 g of dry matter). It was confirmed that the main component among flavonoids (in total amounts reaching up to 1394 mg per 100 g of dry matter) in chokeberry fruit is quercetin and quercetin glycosides, such as quercetin-3-galactoside, quercetin-3-glucoside and quercetin-3-rutoside. On the other hand, among the anthocyanins in chokeberry fruit, the presence of cyanidin glycosides, such as cyanidin-3-arabinoside, cyanidin-3-galactoside, cyanidin-3-glucoside and cyanidin-3-xyloside, among others, was confirmed. Among the phenolic acids, the presence of chlorogenic acid and neochlorogenic acid was confirmed, which account for about 7.5% of the polyphenolic compounds in chokeberry fruit [47].

It has been observed that the polyphenolic components of red wine (when consumed in non-alcoholic or low-alcohol form) can prevent increases in blood pressure, including when these increases are a result of hyperglycaemia or diabetes, which often lead to oxidative damage and vascular dysfunction. In animal model studies (hypertensive rats), the stilbene components of red wine have been shown to increase the antioxidant capacity of blood and vascular endothelial cells. Resveratrol has been confirmed to lower blood pressure by decreasing the activity of angiotensin II and β1-adrenergic receptors located in the heart and kidneys [84]. By inhibiting β1-adrenergic receptors located in cells of the renal glomerular apparatus, resveratrol attenuates the secretion of renin, the enzyme that is the first component of the renin–angiotensin–aldosterone system (RAAS). Inhibition of RAAS activation leads to vasodilation of blood vessels and reduced water and sodium retention, which consequently lowers blood pressure [97].

A study conducted on an animal model (rats) confirmed that polyphenolic compounds from the seeds of Malabar cardamom (*Elettaria cardamomum* L. Maton) can also reduce hypertension associated with metabolic syndrome, which is linked to a high-calorie diet rich in simple sugars accompanied by low physical activity. Consumption of flavonoid-rich cardamom flower and seed extract, despite high fructose intake, has been confirmed to lower blood pressure, as well as normalise the lipid profile (total cholesterol, HDL and LDL cholesterol and triglyceride levels) and blood glucose levels. It has been observed that the hypotensive effect of cardamom phenolic extract occurs by increasing the efficiency of nitric oxide (NO) formation, resulting in increased levels of NO in the blood and consequently improved vascular function. Phenolic acids such as protocatechuic acid, caffeic acid, syringic acid and 5-O caffeoylquinic acid are mainly responsible for the observed vasodilatory effect [39].

Results from an animal model (C57BL/6 mice) showed that consumption of small berries such as blackberry (*Rubus* L.) and raspberry (*Rubus idaeus* L.) can alleviate the effects of hypertension caused by a diet rich in fat and sucrose [52]. A high-carbohydrate, high-fat diet has been confirmed to result in the overproduction of reactive oxygen species (ROS). This occurs because excessive fat consumption triggers intensive mitochondrial β-oxidation, leading to increased production and accumulation of ROS [98].

In addition, increased expression of NADPH oxidase (NOX) has been observed in cardiomyocytes, adipocytes, and skeletal muscle in obese organisms. The activation and increased expression of NOX in the cardiovascular system is associated with the intense production of reactive oxygen species (ROS), mainly O_2_^∙-^ and H_2_O_2_. The ROS thus produced in the vascular endothelium react with nitric oxide (NO) to form peroxynitrite (ONOO^-^), which reduces the bioavailability of endothelial nitric oxide (NO). ONOO^-^ is a potent oxidant with cytotoxic effects, which causes vascular endothelial cell dysfunction [52].

A high-carbohydrate and high-fat diet has also been shown to increase angiotensin type 1 receptor (AT1R) expression in the kidney and aorta. AT1R activation results in increased renal sodium reabsorption and increased vasoconstriction, which consequently elevates blood pressure. Phenolic compounds in raspberry and blackberry fruit (especially when consumed together) may increase the bioavailability of endothelium-derived nitric oxide by attenuating AT1R receptor expression in the kidney and arterial vessels. In addition, it has been shown that even with a high intake of palmitic acid, the consumption of blackberry and raspberry fruit causes (3.9 and 3.8-fold, respectively) an increase in the amount of Nrf2 in human aortic endothelial cells. Phenolic compounds from blackberry and raspberry fruit can promote Nrf2 activation and consequently lead to dephosphorylation of phospho-eNOS, activating nitric oxide synthesis, resulting in vasodilation and blood pressure reduction [52].

Analyses have shown that the antihypertensive properties of blackberry fruit are directly related to polyphenolic compounds such as ellagotannins, cyanidin-3-glucoside and quercetin [99]. In raspberry fruit, vasodilatory properties were attributed to phenolic compounds such as catechin; epicatechin; procyanidin B4; flavan-3-ol trimer; quercetin; quercetin-3-O-glucuronide; lambertianin C; sanguiin H-6; cyanidin-3-O-sophoroside; cyanidin-3-O-sambubiside; cyanidin 3-O-glucoside; and cyanidin-3-O-rutinoside [52,53].

It has been shown that standardised tea extracts, such as white tea extract and black and green tea extracts, can have therapeutic applications and significantly reduce the risk of cardiovascular disease. In an animal model study (C57/BL6J mice) that received a high-fat, high-saccharose diet for 20 weeks, the animals developed metabolic syndrome and obesity, along with a significant increase in blood pressure due to a significant decrease in endothelial nitric oxide synthase (eNOS) gene expression. It was also observed that obesity is significantly associated with an increase in IL-1β, IL-6, and NOX-4 gene expression in aortic wall tissue [63]. Interleukin 1 beta (IL-1β) and Interleukin 6 (IL-6) are pro-inflammatory cytokines that can lead to vascular endothelial damage and atherosclerotic plaque formation [100]. In turn, NADPH oxidase NOX-4 is a source of reactive oxygen species within arterial blood vessels [90], and, through hypertrophy of vascular cells, affects vasoconstriction [101].

On the other hand, it has been observed that obesity is significantly associated with decreased mRNA levels of the antioxidant enzymes GPX-3 and SOD-1 in aortic wall cells [90]. In contrast, supplementation with both white tea extract and black and green tea extracts can significantly reduce obesity-induced elevated blood pressure. The beneficial antihypertensive effects of black and green tea extract were related to the presence of gallic acid, flavan-3-ols, and (-)-epigallocatechin-3-gallate. The therapeutic effect of these compounds is manifested by an increase in the secretion of vasoactive substance, such as nitric oxide (NO), resulting in improved vascular endothelial function [63].

It has been confirmed that the polyphenolic extract of Southern cattail (*Typha domingensis* Pers.), may also exhibit potent antihypertensive properties, especially when the established hypertension is the result of existing hyperlipidaemia induced by a high-calorie, high-carbohydrate and high-fat diet. It has been indicated that the therapeutic effect of Southern cattail is due to the presence of flavonoid glycosides such as hyperoside (a quercetin glycoside), quercetin rhamno-di-hexoside and quercetin-3-O-glucopyranoside, flavanones such as naringenin and phenolic acid glycosides such as chlorogenic acid and ferulic acid. Ferulic acid contained in Typha domingensis exhibits antioxidant properties and potent hypotensive effects, which are associated with activation of inducible nitric oxide synthase in vascular smooth muscle cells, resulting in increased nitric oxide synthesis and vasodilation. In animal model studies (Wistar rats with spontaneous hypertension), it has been shown that the polyphenolic bioactive compounds present in *Typha domingensis* can lower blood pressure (accompanying hyperlipidaemia) also through a mechanism of increasing the volume of urine excreted from the body and a mechanism of increasing the excretion of excess sodium ions (Na^+^) in the urine, leading to a reduction in blood pressure [42].

It has been confirmed that the polyphenolic bioactive compounds contained in the leaves and bark of the Madagascar almond tree (*Terminalia neotaliala*) can effectively support the treatment of hypertension. The described vasodilatory effect may be due to the presence of phenolic acids (such as gallic acid, quinic acid, ellagic acid), gallic acid esters (such as methyl gallate), phenolic coumarins (such as scopoletin), flavonols (such as quercetin) and hydrolysable tannins (such as ellagitannins and gallitannins). Polyphenolic bioactive compounds in almond tree leaf and bark extract have also been confirmed to exhibit hypoglycaemic properties, acting similarly to α-glucosidase inhibitors. Polyphenols from *T. neotaliala* inhibit α-glucosidase activity in the epithelium of the small intestine, thereby effectively delaying postprandial hyperglycaemia. The antidiabetic effect is also due to the ability of the hydrolysable tannins to block glucose uptake by the intestinal epithelium and increase glycogen synthesis. For this reason, an extract derived from the leaves and bark of the almond tree can be used in people with hypertension who additionally suffer from type II diabetes [45].

The results of the study indicate that the polyphenols contained in the petal extract of the large-flowered rose (*Rosa Tifola*), may also exhibit vasorelaxant effects. In studies on isolated aorta, phenolic compounds such as quercetin rutoside, quercetin and protocatechuic acid contained in *Rosa Tifola* were confirmed to effectively prevent dysfunction of human vascular endothelial cells. The observed vascular relaxation is due to the activation of nitric oxide synthase within the arterial endothelium by the phenolic compounds, and is also associated with the activation of adenylate cyclase and the opening of potassium channels. In vascular wall smooth muscle cells, the opening of potassium channels causes an efflux of potassium ions (K^+^) from the cell, resulting in the closure of the potential-dependent calcium channels (L-type) and the consequent blocking of the influx of calcium ions (Ca^2+^) into the cell. This reduced intracellular calcium ion concentration leads to relaxation of smooth muscle cells, resulting in vasodilation [61].

Polyphenols (including quercetin rutoside, quercetin, epigallocatechin gallate, kemferol, luteolin, naringenin, gallic acid, chlorogenic acid, vanillic acid and protocatechuic acid) have also been shown to have an anti-inflammatory effect on endothelial cells, by inhibiting the activity of tumour necrosis factor alpha (TNF-α) and nuclear factor kappa B (NF-κB), which in vascular endothelial cells activates the expression of genes for pro-inflammatory cytokines [61]. For this reason, polyphenols from *Rosa Tifola* petals (primarily such as rutin, quercetin and protocatechuic acid) may provide important protection for the vascular endothelium, especially when exposed to the effects of a high-monocarbohydrate diet (glucose) and consequently to the effects of high production of reactive oxygen species in the body [61].

It has been shown that *Nigella sativa* seed extract can be effective in lowering blood pressure in people with mild hypertension. The phenolic compounds contained in *N. sativa* seeds can reduce systolic blood pressure by an average of 3.26 mmHg and diastolic blood pressure by 2.80 mmHg in mild hypertension, also in people with established type 2 diabetes. The described effect was obtained when study patients consumed *Nigella sativa* seeds orally at 2 g/day for 12 months. It is suggested that the antihypertensive mechanism of polyphenols from *N. sativa* is due to the antioxidant activity of flavonoids present in its seeds, such as quercetin, kemferol and amentoflavone [102]. The mechanism is also attributed to their ability to stimulate endothelial nitric oxide synthase (eNOS) activity, resulting in increased nitric oxide production and vasodilation [50].

It has been shown that an extract of oscomiana bilimbi (*Averrhoa bilimbi* L.) may be effective against hypertension, particularly when induced by oxygen free radicals as a result of ethanol intoxication [103]. When consumed in large quantities, alcohol initially causes a short-term reduction in blood pressure due to vasodilation, followed by a compensatory increase, leading to detrimental pressure fluctuation [104]. Based on studies in an animal model (male Wistar rats), it has been shown that polyphenols, including flavonoids from the fruit extract of oscomiana bilimbi, can increase serum nitric oxide (NO) concentrations and thereby cause vasodilation in hypertension induced by several days’ (15 days) consumption of 30% ethanol. Histopathological findings have shown that blood vessels of ethanol-treated animals become inflamed within the vascular capillary membrane and show degradation of the endothelium, hydropic degeneration, and the presence of necrotic cells in the inner membrane of blood vessels [103]. Formed as a result of ethanol metabolism, acetic acid induces oxidative stress and the formation of large amounts of oxygen free radicals, which inhibit the activity of endothelial eNOS synthase and consequently inhibit NO formation, leading to cardiovascular dysfunction. Histopathological analysis has shown that polyphenols in *A. bilimbi* L. extract can limit the breakdown of endothelial cells and cells of the inner layer of blood vessels, thereby reducing vascular dysfunction. Polyphenols in the extract may also reduce leukocyte infiltration into the appendage. It has been confirmed that flavonoids from the fruit extract may (indirectly through the protection of endothelial NO synthase and NO synthesis) have an important function not only in vasodilation, but also a protective function, acting as anti-thrombotic compounds that inhibit leukocyte adhesion, monocyte migration, and the secretion of pro-inflammatory cytokines [103].

### 3.4. Polyphenols in the Treatment of Hypertension Related to Chronic Kidney Disease

Findings suggest that flavonoids may also be effective in lowering blood pressure in people with increased arterial stiffness, such as patients with chronic kidney disease (CKD), stage I-IV. It has been confirmed that daily intake of flavonoids (200 mg) from raw materials such as cocoa beans (*Theobroma cacao* L.), lemon balm leaves (*Melissa officinalis* L.), purple leaves and flowers (*Cistus incannus*), or pomegranate fruit (*Punica* L.) for a period of 3 months can reduce the carotid artery pulse wave velocity and lower peripheral systolic blood pressure by 14 mm Hg. Flavonoid consumption can also reduce carotid pulse wave velocity from 8.9 m/s to 8.2 m/s and significantly reduce central arterial pressure from 59 mm Hg to 48 mm Hg [58]. It has been suggested that the therapeutic effects are due to the presence in the raw materials studied of large amounts of flavonoids such as kemferol and quercetin, which, due to their anti-inflammatory and antioxidant effects, reduce the inflammation and oxidation induced by angiotensin II [58,105]. Furthermore, a reduction in oxidative stress markers in the form of reduced blood carbonyl protein (from 73.50 nmol/mL to 52.54 nmol/mL) was confirmed as a result of daily consumption of increased amounts of flavonoids [58].

It has been confirmed that the seed extract of the subtropical plant, the Chinese lychee (*Lychee chinensis* Sonn.), can reduce hypertension and protect against hypertensive kidney damage. A study in 12-week-old male rats with spontaneous hypertension (SHR) showed that polyphenols present in lychee seeds can inhibit the TNF signalling pathway, in which TNF-α is a key mediator of inflammation, whose excessive activation contributes to increased blood pressure [44]. TNF-α can activate the renin–angiotensin–aldosterone system (RAA system), leading to increased expression of angiotensin II, a potent vasoconstrictor that also causes sodium and water retention [106].

**Figure 9:**
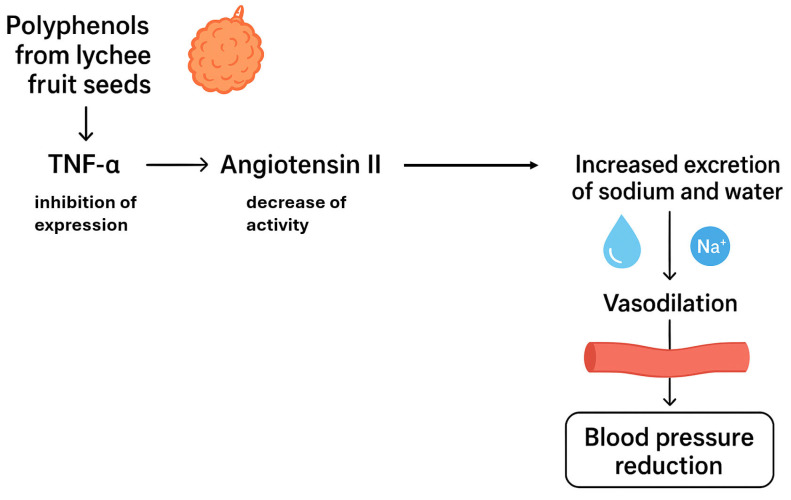
Basic mechanism of vasodilatory action of phenolic compounds from Chinese lychee (*Lychee chinensis* Sonn.) [44,106].

Figure 9 graphically presents the mechanism of vasodilatory action of phenolic compounds derived from Chinese lychee by inhibiting the activity of the renin–angiotensin–aldosterone system (RAA system).

Using network pharmacological analysis, it was shown that the therapeutic effect of polyphenolic compounds from the seeds of *L. chinensis* Sonn. may also be related to an inhibitory effect on the interleukin-6 (IL-6)-dependent signalling pathway, which is a potent pro-inflammatory factor [44]. Elevated levels of IL-6 in hypertension lead to chronic inflammation within the blood vessel walls, causing endothelial damage and reduced nitric oxide (NO) production. In addition, polyphenols from lychee fruit seeds have also been observed to inhibit the NF-κB signalling pathway, one of the main mediators of inflammation that activates the production of pro-inflammatory cytokines such as TNF-α. This induces chronic inflammation, contributing to increased blood pressure [107].

**Figure 10:**
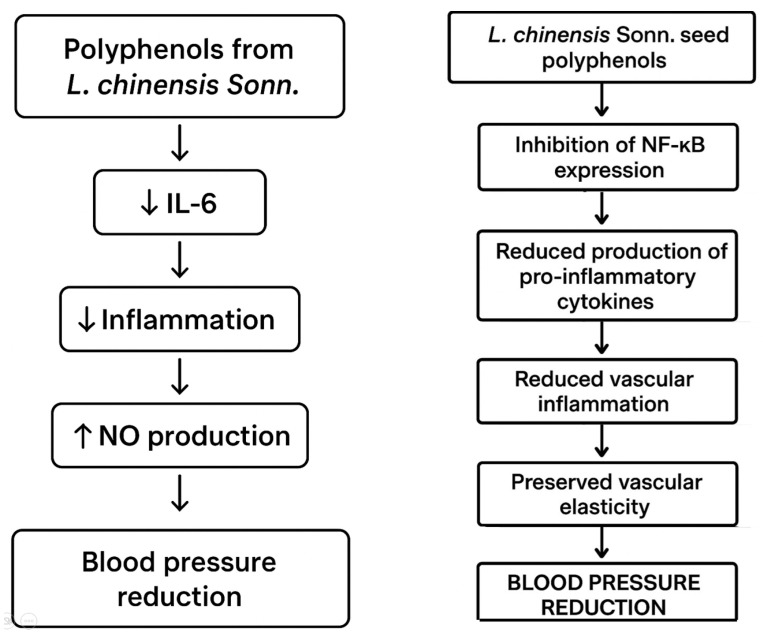
Mechanisms of vasodilatory action of phenolic compounds derived from Chinese lychee (*Lychee chinensis* Sonn.) by reducing the level of IL-6 and inhibiting the expression of NF-κB factor [44,106,107].

Figure 10 graphically presents additional mechanisms of vasodilatory action of phenolic compounds derived from Chinese lychee by reducing the level of IL-6 and inhibiting the expression of NF-κB factor.

Furthermore, it has been observed that the consumption of polyphenols from *L. chinensis* Sonn seeds in the presence of spontaneous hypertension protects kidney cells from hypertension-induced damage, mainly by inhibiting inflammation and oxidative stress. It is indicated that polyphenols identified in Chinese lychee seeds, such as procyanidin B2, epicatechin, cinnamtannin B1, cinnamtannin B2, an isomer of cinnamtannin B1, procyanidin A1, quercetin, rutin, procyanidindimer A, phlorizin, aesculitannin C and cyanidin-3-glucoside, are responsible for the observed antihypertensive effects. The consumption of polyphenols from lychee fruit seeds can significantly increase the relative abundance of *Lactobacillus*, *Turicibacter* and *Romboutsia* bacteria in the gastrointestinal tract and consequently the production of short-chain fatty acids (SCFAs), mainly butyric acid. The SCFAs produced by intestinal bacteria, such as butyric acid, acetic acid and propionic acid, when absorbed into the bloodstream, can act on pro-renin receptors in blood vessel walls [44]. Sodium butyrate has been confirmed to inhibit renal pro-renin receptor activity and the intrarenal renin-angiotensin system, thereby reducing angiotensin II-induced hypertension [108].

It has been shown that quercetin, known for its general antihypertensive effect, can also protect and prevent hypertensive kidney damage. A study in an animal model confirmed that quercetin consumption can significantly alleviate pathological changes in renal interstitial tissue and renal tubule cells when angiotensinogen is converted to angiotensin II. Quercetin was shown to inhibit signalling pathways associated with cell apoptosis in renal interstitial tissue and renal tubular cells. Molecular docking analysis indicates a potential binding interaction between the quercetin molecule and the TP53 gene, which encodes the multifunctional protein p53. Quercetin, by inhibiting the function of p53 protein, may prevent cell apoptosis within the kidney. Quercetin consumption also inhibits the expression of the Bax protein and causes inhibition of cleaved caspase-9 and cleaved caspase-3. Therefore, consumption of quercetin can significantly reduce renal damage caused by hypertension and inhibit cell apoptosis in renal tissues when angiotensin II activity is high in the body [109].

It has been confirmed that quercetin may have antihypertensive and protective effects in the prevention of cardiovascular disease also through its effect on arachidonic acid metabolism. In an experiment using an animal model (spontaneously hypertensive rats), it was shown that the consumption of quercetin can effectively counteract the development of hypertension. Clinical studies have confirmed that arachidonic acid metabolites, such as hydroxyeicosatetraenoic acids (HETEs), formed with cytochrome CYP4A, cause constriction of peripheral blood vessels. Polyphenols such as quercetin can inhibit the activity of cytochrome CYP4A (among others in renal cortex microsomes), thereby reducing the amount of HETEs formed. Furthermore, it has been shown that higher doses of quercetin can significantly inhibit the degradation of bioactive epoxyeicosatrienoic acids (EETs), which have strong vasodilatory properties. EETs are degraded in the cytosol of renal cortex cells by the enzyme epoxide hydrolase. Quercetin is able to inhibit the activity of epoxide hydrolase, thus preserving the vasodilatory capacity of EETs [110]. EETs are strong vasodilators, mainly due to their ability to activate K^+^ channels and modulate angiotensin II activity. Angiotensin II causes constriction of small arterioles, which directly increases vascular resistance and raises blood pressure [111]. Therefore, the increased bioavailability of EETs in the body as a result of quercetin consumption may stimulate the body to lower blood pressure [110].

It has also been confirmed that apigenin, a commonly present flavonoid, can inhibit the formation of hydroxyeicosatetraenoic acid (HETEs) as a result of blocking the ω-hydroxylation of arachidonic acid by cytochrome P450 monooxygenase. Thus, by inhibiting the formation of a potent vasoconstrictor, this polyphenol contributes to lowering blood pressure [112].

Resveratrol has been confirmed to counteract changes in renal function and structure in malignant hypertension. In animal model studies (in rats), resveratrol has been shown to prevent long-term renal damage resulting from high blood pressure, in the form of degradation and reduction in podocytes, the specialised visceral epithelial cells of the glomeruli. Resveratrol also preserves glomerular volume, which is significantly reduced as a result of high blood pressure [59].

Table 2 presents the biochemical mechanisms of action of extracts and phenolic compounds with antihypertensive properties.

### 3.5. Limitations of Polyphenolic Compounds in Hypertension Treatment

The choice of a specific polyphenolic substance (or plant material rich in polyphenols) for treating or preventing hypertension should be largely an individual matter and depend on the cause of the high blood pressure.

When using polyphenols, potential contraindications to consuming a given polyphenolic substance should be taken into account. Although polyphenolic compounds are generally considered safe, it should be noted that polyphenols may cause allergic reactions in some people, including allergic contact and irritant dermatitis and mucosal inflammation [113]. This risk stems from the ability of certain polyphenols (such as phloretin) to react with skin proteins in a Michael addition reaction, generating an antigen that leads to an immune response [114].

There is also a general risk associated with the interaction of a given polyphenolic substance with medications taken for hypertension or other diseases. It has been shown that isoflavones (such as genistein, glycitein and daidzein) can compete with thyroxine (T4) at the site of attachment to transthyretin (the main transport protein for thyroid hormones in the blood). Therefore, the consumption of large amounts of isoflavones by people with thyroid disorders who are undergoing hormone treatment may cause a decrease in the concentration of free thyroid hormones in tissues, leading to impaired fat metabolism, weakened carbohydrate metabolism, and digestive system function [115].

It has also been pointed out that consuming foods rich in certain polyphenolic compounds can significantly interfere with the absorption, distribution, and metabolism of medications [115]. It has been confirmed that polyphenolic compounds such as hesperidin, rutin and apigenin can inhibit the activity of cytochromes P450 (CYP450), a large and diverse group of enzymes produced mainly in the liver, one of whose functions is the biotransformation of drugs [112,116]. Thus, certain polyphenolic compounds may affect the half-life of drugs, altering their efficacy and increasing their potential side effects. Therefore, polyphenolic compounds that could inhibit the activity of certain forms of CYP450, such as CYP3A4, should be excluded [115,116].

In addition, some polyphenols may also adversely affect drug transport by, among other things, interacting with transport proteins found in cell membranes [115]. An example is the interaction of certain catechins, such as epicatechin gallate, with P-glycoprotein, a multidrug resistance protein (MDR1) known as an efflux pump [117]. By binding to P-glycoprotein, catechins can reduce the activity of the efflux pump and thus alter the effectiveness of drug treatment [117].

Epigallocatechin gallate (EGCG), found in green tea extract, among others, has also been shown to increase the risk of liver damage when consumed in high doses. It has been suggested that certain consumer groups (e.g., postmenopausal women) may exhibit reduced activity of catechol-O-methyltransferase (COMT) and uridine-5′-diphosphoglucuronosyltransferase 1A4 (UGT1A4), leading to slower EGCG metabolism and, as a result, prolonged liver exposure to potentially toxic levels of catechin [118].

When consuming specific polyphenolic compounds, one should also take into account one’s overall health and other characteristics of the body, such as age, body weight, or the presence of other chronic diseases. It should be borne in mind that age-related changes in digestion, absorption and the composition of the gut microbiota may adversely affect the bioavailability of polyphenolic compounds, thereby reducing their therapeutic efficacy in relation to hypertension [119].

The gut microbiota plays a key role in metabolising polyphenols and converting them into bioactive forms [120]. In older people and those with chronic diseases such as irritable bowel syndrome or inflammatory bowel disease, the composition and functioning of the gut microbiota may be disrupted. This, in turn, can significantly reduce the body’s absorption of micronutrients, including polyphenols [121,122].

The results presented in this article suggest that the goal of therapy with bioactive polyphenolic compounds should be to lower blood pressure to a value close to 140/90, which is recommended by cardiologists and cardiology organisations [4]. The cited research results also indicate that bioactive polyphenolic compounds supplementing the diet should cause a significant reduction in blood pressure within 4–6 weeks of starting their use [84,96].

Research results indicate that preventing and treating hypertension not only protects blood vessels from damage, but also reduces the risk of heart attack and other cardiovascular incidents, such as stroke or kidney failure. Some types of polyphenols, such as honokiol, have both antihypertensive effects and protect the brain by activating pathways and mechanisms associated with anticoagulant activity [123]. In turn, polyphenolic compounds such as kaempferol, quercetin and resveratrol, in addition to their antihypertensive effects, also prevent oxidative stress in heart tissues and inhibit the expression of inflammatory markers in cardiomyocytes, thus preventing inflammatory processes in the heart muscle. Consequently, consuming these polyphenols may prevent heart diseases such as heart failure and ischaemic heart disease [124].

Effective treatment and prevention of hypertension is particularly important due to the projected increase in mortality among populations on various continents, especially in Europe and North America. Available data indicate that immigrant populations arriving in Europe and North America are characterised by a significantly higher risk of cardiovascular disease compared to the populations of the host countries. This is influenced by factors such as the lower socio-economic status of immigrants, unfavourable lifestyle, psychological stress, and limited access to healthcare. This means that as the length of stay in the new country increases, immigrants very often experience a deterioration in their cardiovascular health. The initial, often better physical and health condition of immigrants, referred to as the “healthy migrant effect”, disappears over time, which ultimately contributes to an increased incidence of cardiovascular diseases, including hypertension [125].

An important element of antihypertensive therapy and prevention is also the patient’s high level of involvement in the treatment process [126]. This process should include not only a better understanding of the condition itself, i.e., hypertension and its consequences, but also an understanding of the antihypertensive effects of polyphenolic compounds. It has been confirmed that the more knowledge the consumer (patient) has about the benefits of therapy, the higher their participation in therapy and the greater their compliance with medical and dietary recommendations [126].

The scientific reports presented suggest that methods of treating and preventing hypertension based on the consumption of polyphenolic compounds contained in natural, unprocessed plant materials may be an effective method of preventing this disease. The consumption of polyphenolic compounds is particularly important for people over the age of 60 [52]. It has been confirmed that with age, the amount of peroxynitrite (ONOO^-^) increases. Peroxynitrite is a highly reactive nitrating oxidant that also captures and degrades nitric oxide (NO) molecules, exacerbating blood vessel dysfunction with age, which in turn leads to even higher blood pressure [68].

Numerous studies indicate that the treatment of hypertension should be supplemented with regular physical activity, which can significantly reduce systolic blood pressure (by as much as 4–9 mm Hg) and thus reduce the risk of blood vessel damage [127,128].

It is recommended that people with diagnosed hypertension perform moderately intense but dynamic aerobic exercises (such as walking, jogging, cycling or swimming) for at least 30 min, 5–7 days a week. In addition, it is recommended that aerobic exercise be supplemented with additional resistance (muscular) training 2–3 days a week, as the combination of both has been shown to be highly effective in lowering blood pressure [129].

Antihypertensive therapy and prevention using polyphenolic compounds should be supplemented with additional measures that reduce the risk of cardiovascular disease. The most important of these recommendations are weight normalisation and fat reduction, and a properly balanced diet based on increased consumption of unprocessed vegetables and fruit, while limiting highly processed foods rich in saturated fats [130,131,132].

It has been observed that consuming 800 g of vegetables per day and 550 g of fruit per day can significantly reduce the risk of hypertension [132]. In preventing and reducing hypertension, it is also important to limit sodium intake (for adults to less than 2 g sodium/day), reduce alcohol consumption (to less than 12 g of alcohol per day) and stop using tobacco products [133,134,135]. It has been confirmed that combining tobacco and alcohol consumption is a particularly harmful habit, significantly increasing the long-term risk of hypertension [135].

Table 3 presents selected recommendations regarding the consumption of phenolic compounds by people with a tendency to hypertension, which were developed on the basis of scientific research results.

## 4. Conclusions

An analysis of scientific articles on the significance and impact of phenolic compounds in the prevention and treatment of hypertension has led to the general conclusion that flavonoids are a group of polyphenols characterised by effective and multifaceted antihypertensive therapeutic effects. Based on the research results described in this review, it can be concluded that natural phenolic compounds may be effective in counteracting hypertension and at the same time their consumption is safe and in the vast majority of cases does not cause side effects.

The cited research results clearly indicate that consuming natural phenolic compounds may also be an important supplement to traditional pharmaceutical therapy for hypertension. At the same time, based on the knowledge gathered in this review, it should be noted that data on the effects of certain phenolic compounds in terms of their absorption, distribution and metabolism are still very limited, and thorough pharmacodynamic studies of many of these compounds have not yet been undertaken to a sufficient extent.

Future research on polyphenols should focus on examining in even greater detail the molecular and biochemical mechanisms of action of individual phenolic compounds in the prevention of hypertension, and on empirically determining the exact amounts of polyphenols consumed that can provide the best therapeutic and health benefits. The coming years should bring new scientific discoveries and interesting information on the prevention and treatment of various types of hypertension using already known phenolic compounds, as well as completely new, yet undiscovered polyphenols.

## Figures and Tables

**Table 1:** Phenolic compounds with antihypertensive effect and their source of origin.

Phenolic Component	Type (Group) of Phenolic Compound	Source/Raw Material (Common Name/ Latin Name)	Part of Plant	Type of Biological Activity	Ref.
3,5-di-o-galloylquinic acid, gallic acid	phenolic acids	the mastic pistachio (*Pistacia lentiscus* L.)	leaves	antioxidant, anti-inflammatory	[38]
protocatechuic acid, caffeic acid, syringic acid, 5-o-caffeoylquinic acid	phenolic acids	malabar cardamom (*Elettaria cardamomum* L.) maton)	flowers/seeds	antioxidant, anti-inflammatory, hypoglycemic effect, hypolipidemic effect	[39]
myricetin	flavonol	black mangrove/mangrove plant, (*Lumnitzera racemosa*)	leaves	antioxidant, anti-inflammatory,	[40]
rosmarinic acid	phenolic acids				
4-ethenyl-2,6-dimethoxy-phenol	metabolic product (derivative) of ferulic acid	cabbage (*Brassica rapa*)	leaves	antihypertensive, anti-inflammatory	[41]
quercetin hyperoside-rhamno-di-hexoside, quercetin-3-o-glucopyranoside	quercetin glycosides	southern cattail (*Typha domingensis* pers.)	young shoots/leaves	antihypertensive antioxidant diuretic stimulating the excretion of excess sodium ions, preventing fatty liver disease	[42]
naringenin	flavanone				
chlorogenic acid glycoside, ferulic acid glycoside	phenolic acid glycoside				
myricetin rhamnoside	flavonol	*Flemingia faginea* Guill. & Perr.	leafy stems	antihypertensive antioxidant	[43]
myricetin rutinoside					
quercetin rutinoside,					
caffeoyl glucoside	derivatives apigenin				
5-caffeoylquinic acid (chlorogenic acid)	phenolic acids				
gallic acid glycoside	phenolic acid glycoside				
procyanidin b2, cinnamtannin b1, cinnamtannin b2, procyanidin a1, procyanidindimer a	proanthocyanidin	lychee (*Litchi chinensis* Sonn.)	seeds	antihypertensive antioxidant anti-inflammatory diuretic	[44]
epicatechin	fla-van-3-ol				
quercetin	flavonol				
quercetin-3-rutinoside (rutin)	flavonoid glycoside				
phlorizin	dihydrochalcone				
aesculitannin c	tannin				
cyanidin-3-glucoside	anthocyanin				
procyanidin b2 cinnamtannin b1 cinnamtannin b2 procyanidin a1 procyanidindimer a	proanthocyanidin	Madagascar almond (*Terminalia neotaliala*)	leaves/bark	antihypertensive, hypoglycemic	[45]
epicatechin	flavanol				
quercetin	flavonol				
quercetin-3-rutinoside (rutin)	flavonoid glycoside				
phlorizin	dihydrochalcone				
aesculitannin c	tannin				
cyanidin-3-glucoside	anthocyanin				
chlorogenic acid, syringic acid, sinapic acid	phenolic acid	watermelon coloquinta (*Citrullus colocynthis*)	fruit	antihypertensive antioxidant cardioprotective	[46]
quercetin-3-rutinoside (rutin)	flavonoid glycoside				
kaempferol-3-glucoside, myricetin-3-o-glucuronide	flavonol				
resveratrol	stilben				
chlorogenic acid, syringic acid, sinapic acid	phenolic acids	black chokeberry (*Aronia melanocarpa*)	fruit	antihypertensive antioxidant prevents metabolic syndrome antidiabetic	[47]
kaempferol-3-glucoside, quercetin-3-rutinoside (rutin)	flavonoid glycoside				
myricetin-3-o-glucuronide	flavonoid glucuronide				
resveratrol	stilben				
catechin, catechin gallate, epicatechin	flavanols	muscat grapes (*Vitis vinifera*)	skins/seeds	antihypertensive, antioxidant, antiperoxidant	[48]
tiliroside	flavonol derivative	*Alchemilla viridiflora* Rothm., *Rosaceae*	leaves, flowers	antihypertensive antioxidant	[49]
pentose ellagic acid galloyl-hexahydroxydiphenyl-glucose	phenolic acid derivative				
miquelianin	flavonol glucuronide				
quercetin	flavone	black cumin, *Nigella sativa*	seeds	antihypertensive antioxidant	[50]
kempferol	flavonone				
amentoflavone	biflavone				
neoeriocitrin, neohesperidin, naringin, bruteridine, melitidine	flavonoid glycoside	cultivations of citrus Bergamia Risso & Poiteau	bergamot juice	antihypertensive, antioxidant, vasculogenic (angiogenic), regenerating vascular cells	[51]
Catechin, epicatechin	flavan-3-ol	raspberry (*Rubus idaeus* L.)	fruit	antihypertensive antioxidant	[52,53]
procyanidin b4	catechin-(4α→8)-epicatechin dimer				
Quercetin, quercetin-3-o-glucuronide (miquelianin)	flavonol				
lambertianin c, sanguiin h-6	ellagitannin				
cyanidin-3-o-sophoroside, cyanidin-3-o-sambubiside, cyanidin-3-o-glucoside, cyanidin-3-o-rutinoside	anthocyanins				
cyanidin-3-o-arabinoside, cyanidin-3-o-galactoside, malvidin-3-o-arabinoside, malvidin-3-o-galactoside, malvidin-3-o-glucoside, paeonidin-3-o-galactoside, paeonidin-3-o-glucoside, petunidin-3-o-galactoside	anthocyanins	highbush blueberry (*Vaccinium corymbosum*), variety tifblue, wild lowbush blueberry (*Vaccinium virgatum*), variety rubel	fruit	antihypertensive antioxidant anti-inflammatory	[54,55]
patuletin-7-(6′’-(2-methylbutyryl)-glucoside)	acylated flavonol	umgabunkhomo plant (*Carissa edulis* Vahl.), the “akan-nsisiri” plant, (*Diodia scandens* sw.), the plant “African spider flower” (*Cleome gynandra* L.),	leaves	antihypertensive, antioxidant	[56]
catechin 3-o-rutinoside	flavan-3-ols				
6-c-glucosylquercetin	flavonoid-3-o-glycosides				
pinocembrin-7-o-rutinoside	flavanone derivative	*Ziziphora clinopodioides subsp. bungeana* (Juz.) Rech.f.	leaves	antihypertensive antioxidant	[57]
chrysin-7-o-rutinoside (5,7-dihydroxyflavone)	flavone derivative				
acacetin-7-o-rutinoside	flavone derivative				
luteolin-7-o-rutinoside	flavone derivative				
quercetin-3-rutinoside (rutin)	flavonoid glycoside				
kaempferol quercetin	flavonol	cocoa (*Theobroma cacao* L.)	seeds	antihypertensive antioxidant anti-inflammatory lowering pulse wave velocity	[58]
		lemon balm (*Melissa officinalis* L.)	leaves		
		cistus /Greek rock rose (*Cistus incannus*)	leaves/flowers		
		pomegranate (*Punica granatum* L.)	fruit		
trans-resweratrol	3,4′,5-trihydroxystilbene	grapevine (*Vitis vinifera*)	grape skin	antihypertensive, antioxidant, anti-inflammatory, angiogenic, renoprotective, podocyte protective, proteinuria reducing	[59]
catechin, epicatechin	flavan-3-ol	cabernet grape, garnacha grape, mazuela grape, merlot grape (*Vitis vinifera*)	wine lees	antihypertensive antioxidant anti-inflammatory	[60]
procyanidin b2 dimer, procyanidin iso1 dimer	proanthocyanidin				
quercetin, isorhamnetin	flavonol				
gallic acid	phenolic acid				
trans-resveratrol piceatannol	stilbene				
malvidin-3-glucoside malvidin-(6-acetyl)-3-glucoside malvidin-(6-coumaroyl)-3-glucoside (anthocyanins)	anthocyanins				
quercetin rutoside (rutin), quercetin	flavonol	centifolia rose/“cristata” (*Rosa tifola*)	flower petals	antihypertensive antioxidant anti-inflammatory	[61]
protocatechuic acid	phenolic acid				
gallic acid, transferulic acid	phenolic acid				
quercetin-3-rutoside (rutin), iso-quercitrin, quercetin	flavonol	Tuscan wheat spelt (*Triticum dicoccum*)	flour	antihypertensive antioxidant anti-inflammatory	[62]
gallic acid	phenolic acid	white tea/ green tea/ black tea/ (*Camellia sinensis*)	leaf buds/leaves	antihypertensive antioxidant anti-inflammatory counteracting metabolic syndrome and obesity	[63]
epigallocatechin-3-gallate	flavonol				
quercetin-3-rutoside (rutin), quercetin	flavonol	barberry (*Berberis vulgaris* L.)	fruit	antihypertensive antioxidant anti-inflammatory regenerating vascular cells	[64,65]
cyanidin-3,5-diglucoside, cyanidin-3-glucoside, delphinidin-3,5-diglucoside, petunidin-3-o-β-d-glucoside, pelargonidin-3,5-diglucoside, pelargonidin-3-glucoside	anthocyanins				
ellagic acid, chlorogenic acid, caffeic acid	phenolic acid				

**Table 2:** Biochemical mechanisms of action of extracts and phenolic compounds with antihypertensive properties.

Type of Phenolic Extract or Phenolic Component	Type of Research	Type of Research Model	Male/ Female	Mechanism of Action Mechanism of Action → Obtained Result	Ref.
Polyphenolic extract from *Pistacia lentiscus* L.	in vivo	Albino mice	male/female	Inhibition of NADPH oxidase activity → reduction in inflammation in blood vessels	[38]
Polyphenolic extract from highbush blueberry (*Vaccinium corymbosum* L.) and switchberry (*Vaccinium virgatum*)	ex vivo/ in vivo	Human aortic endothelial cells (HAECs)	-	Decrease in SAPK/JNK and p38 MAPK activity → Inhibition of monocyte, macrophage and T lymphocyte activation → reduction in inflammation. Increase in expression of NRF2 → Activation of SOD1 and NAD(P)H:NQO1 gene expression → increased antioxidant protection in vascular endothelial cells. Increase in expression of heme oxygenase-1 (HO-1, Hmox1) → Reduction in inflammatory processes. Reduction phosphorylation of NF-κB p65 → Reduction of pro-inflammatory gene expression encoding cytokines and chemokines.	[54,79]
Polyphenol extract from the skin and seeds of muscat grapes (*Vitis vinifera* ‘Muscat’)	in vivo	Sprague Dawley rats	male	Reduction in left ventricular filling pressure → improved diastolic function Reduction in cardiomyocyte growth. Reduction in oxidative DNA damage to cardiomyocytes. Increase in expression of SOD1 mRNA and CAT mRNA in the heart → Increased SOD1 and CAT activity in cardiomyocytes.	[48]
Polyphenol extract from *Alchemilla viridiflora* Rothm.	in vitro	ACE Kit-WST	-	Inhibition of angiotensin I-converting enzyme by blocking the enzyme’s active site.	[49]
Polyphenol extract from bergamot fruit (*Citrus bergamia*) of the Risso & Poiteau variety	in vivo/ex vivo	stroke-prone spontaneously hypertensive rats (SHR rats) primary cerebral endothelial cells isolated from newborn SHRSP rat brains	male/female	Reduction in oxidative stress in cerebral vascular endothelial cells → limiting blood vessel damage. Stimulating angiogenesis in the brain. Stimulating endothelial cell migration → accelerating the repair process of damaged vessels.	[51]
polyphenol extract from blackberry (*Rubus* L.) and raspberry (*Rubus idaeus* L.)	in vivo/ in vitro	C57BL/6 mice Human aortic endothelial cells (HAECs)	male	Inhibition of NADPH oxidase expression in cardiomyocytes, adipocytes, and skeletal muscle cells → decreased O_2_^•-^ and H_2_O_2_, ONOO^−^ production → increased nitric oxide bioavailability Weakening of AT1R receptor expression in the kidneys and aorta → decreased renal sodium reabsorption and vasoconstriction → vasodilation Increase in NRF2 levels in aortic endothelial cells → dephosphorylation of nitric oxide synthesis → increased nitric oxide production → vasodilation	[52,53,99]
polyphenol extract from Roselle (*Hibiscus sabdariffa*) and Lemon verbena (*Lippia citriodora*)	in vivo	pre-hypertensive or type 1 hypertensive individuals	male/female	Decrease in angiotensin II activity → reduced blood pressure Increase in adiponectin and PPAR-α expression → reduced cytokine and pro-inflammatory molecule expression → reduced inflammation in blood vessels Reduction in NF-kB (nuclear factor kappa B) protein → reduced cytokine and proinflammatory molecule expression → reduced inflammation in blood vessels	[76]
phenolic extract from the legume *Flemingia faginea* Guill. & Perr.,	ex vivo	mice	male/female	Inhibition of Ca^2+^ ion influx across the cell membrane → reduction in intracellular Ca^2+^ ion concentration → relaxation of blood vessels Release of vascular relaxation mediators (NO, PGI2, and EDHF factor) from the endothelium → reduction in blood pressure	[43]
polyphenol extract from the leaves and bark of the almond tree from Madagascar (*Terminalia neotaliala*)	in vitro	α-Glucosidase from Saccharomyces cerevisiae	-	Scavenging free oxygen radicals from the vascular endothelium → reducing inflammation Inhibition of α-glucosidase activity in the small intestinal epithelium → reducing postprandial hyperglycemia	[45]
polyphenolic wine lees from Cabernet grapes	in vivo	Spontaneously hypertensive rats of the Wistar-Kyoto variety	male/female	Reduction in the activity of angiotensin-converting enzyme → vasodilation	[60]
phenolic extract from: the umgabunkhomo plant (*Carissa edulis* Vahl.), the “akan-nsisiri” plant, *Diodia scandens Sw*., the plant “African spider flower” *Cleome gynandra* L.,	ex vivo	Wistar rats left circumflex porcine coronary arteries and the thoracic aorta	male	Stimulation of the pathway “nitric oxide synthase-NO-guanylate cyclase (guanylate cyclase)-protein kinase G” → relaxation of smooth muscles in blood vessels	[56]
phenolic extract from the leaves of *Ziziphora clinopodioides subsp. bungeana* (Juz.) Rech.f.	ex vivo	Wistar rats isolated mesenteric vascular bed	male	Stimulation of bradykinin B2 receptors on the surface of endothelial cells → vasodilation Activation of the “NO synthase–NO synthesis–guanylate cyclase–protein kinase G” pathway → vasodilation	[57]
phenolic extract from *Malabar cardamom* seeds (*Elettaria cardamomum* (L.) Maton)	in vivo	Sprague-Dawley rats	male	Increase in nitric oxide (NO) production → vasodilation	[39]
phenolic extract from the fruit of the bilimbi plant (*Averrhoa bilimbi* L.)	in vivo	Wistar rats (Rattus norvegicus)	male	Reducing the breakdown of vascular endothelial cells → limiting vascular dysfunction Reduction in leukocyte infiltration into the adventitia → reduction in fibrosis and stiffening of the arterial wall → reducing the risk of hypertension	[103]
phenolic extract from *Rosa Tifola*	in vivo ex vivo	Rats (Wistar strain) Primary Human Umbilical Vein Endothelial Cells isolated from the vein	male	Activation of nitric oxide synthase → increased NO production Activation of adenylate cyclase → opening of potassium channels → efflux of K^+^ ions from the cell → closing of calcium channels (L-type) → blocking the influx of Ca^2+^ ions into the cell → vasodilation Inhibition of TNF-α and NF-κB activity → suppression of pro-inflammatory cytokine gene expression in vascular endothelial cells → reduction in vascular inflammation	[61]
phenolic extract from Tuscan wheat *Triticum dicoccum*	in vitro	Human blood samples The human colonic adenocarcinoma cell (HT-29) line	-	Inhibition of angiotensin-converting enzyme (ACE) activity → inhibition of the conversion of angiotensin I to angiotensin II Reduction expression of the mediator IL-8 → reduced inflammatory processes → reduced expression of adhesion glycoproteins (ICAM-1 and VCAM-1) → reduced adhesion of leukocytes to the blood vessel wall → reduced changes in vessel structure → inhibited progression of atherosclerosis	[62,80]
phenolic extract from shoots and leaves of *Anvillea radiata*	in vivo/ ex vivo	Normotensive rats Isolated aortic rings from rats with functional endothelium	male/female	Inhibition of calcium channel activity → reduced calcium ion influx into vascular smooth muscle cells → vasodilation Stimulation of endothelial nitric oxide synthase (eNOS) → activation of nitric oxide synthesis → vascular smooth muscle relaxation	[81]
phenolic extract from white, black and green tea (*Camellia sinensis*)	in vivo	C57/BL6J mice	male	Increase in secretion of nitric oxide (NO) → vasodilation	[63]
phenolic extract from barberry fruit (*Berberis vulgaris* L.)	in vivo	individuals subjects with hypertension	men/women	Reduction in the amount of macrophage/monocyte chemotactic protein-1 (MCP-1) in the blood → reduction in vascular inflammation Reduction in the amount of VCAM-1 and ICAM-1 glycoproteins in the vessels → reduction in the adhesion and migration of immune cells to the vessel walls → reduction in changes in vessel structure → inhibition of atherosclerosis progression	[64]
phenolic extract from *Brassica rapa* 4-ethenyl-2,6-dimethoxyphenol	in silico/ in vitro	-	-	Angiotensin-converting enzyme I (ACE) inhibition	[41]
3-hydroxyflavone (flavon-3-ol)	in vivo	Swiss albino mice	male	Neutralisation of free oxygen radicals (ROS) → protection of the endothelium of blood vessels	[83]
trans-resveratrol	Gene Ontology (GO) analysis and Kyoto Encyclopedia of Genes and Genomes (KEGG) pathway	-	-	Increase in VEGF expression → stimulation of angiogenesis Inhibition of the sFlt1 receptor (soluble tyrosine kinase) → restoration of angiogenesis Reduction in TNF-α activity → reduced inflammation Reduction in STAT protein activity → reduced inflammation	[87]
trans-resweratrol	in vivo	Spontaneously hypertensive rats (SHR rats)	female	Protection of podocytes (cellular cells of the visceral epithelium of the kidney glomeruli), Inhibition of the production of reactive oxygen species and reactive nitrogen species → lower 3-nitrotyrosine levels → reduced nitrative stress → reduced vascular inflammation Increase in nitric oxide synthase activity → lower blood pressure	[59]
trans-resweratrol	in vivo ex vivo	pregnant rats (Sprague Dawley) isolated endothelial colony-forming cells (ECFCs)	female	Neutralisation of reactive oxygen species → protection of nitric oxide synthase (eNOS) from deactivation Increase in proliferation and capillary formation → increased vascular bed volume → reduced blood flow resistance → lower blood pressure	[94]
trans-resweratrol	in vivo/in vitro	Spontaneously hypertensive rats (SHR rats)	male	Inhibition of β1-adrenergic receptors in the cells of the juxtaglomerular apparatus of the kidneys → weakening of renin secretion → weakening of the activation of the RAAS → reducing water and sodium retention in the body → dilation of blood vessels → lowering of blood pressure	[97]
resveratrol butyrate	in vivo	Hep G2 (a human liver cancer cell line) Virgin Sprague Dawley (SD) rats	female	Reduction in oxidative damage to the kidneys → lower blood pressure Increase in expression of the short-chain fatty acid (SCFA) receptor → activation of FFAR3 (GPR41) receptors in vascular smooth muscle cells by SCFA → vasodilation → lower blood pressure	[92,93]
quercetin	in vivo	Spontaneously hypertensive rats (SHR rats)	male/female	Inhibition of CYP4A activity → reduction in hydroxyeicosatetraenoic acids (vasoconstructors) → reduction in vasoconstriction Inhibition of epoxide hydrolase activity → inhibition of the degradation of bioactive epoxyeicosatrienoic acids (vasodilators) → vasodilation	[110]

**Table 3:** Recommendations for the consumption of phenolic compounds for people with a tendency to hypertension.

Phenolic Product/Supplement	Recommended Polyphenol Dose	Anthropometric Characteristics of Target Consumers	Ref.
phenolic extract from muscat grape skins and seeds (*Vitis vinifera*)	2.3 mg/kg body weight per day	60 kg body weight	[48]
phenolic extract in the form of Cabernet wine less	73 mL per day	70 kg body weight	[60]
black cumin seeds (*Nigella sativa*)	2 g of seeds per day for 12 months	-	[50]
a mixture of flavan-3-ols, anthocyanidins and flavonols	0.235 mg per day	preferably up to 60 years of age	[68]
phenolic extract from hibiscus (*Hibiscus sabdariffa*) flowers and lippia trifoliata (*Lippia citriodora*) leaves	consumed simultaneously 0.175 g of polyphenols from Hibiscus flowers and 0.325 g of polyphenols from Lippia trifoliata leaves	-	[76]
phenolic extract from black chokeberry (*Aronia melanocarpa*)	400 mg of polyphenols per day	-	[47]
phenolic extract from cocoa beans (*Theobroma cacao* L.)	200 mg of polyphenols per day	-	[58]
phenolic extract from lemon balm leaves (*Melissa officinalis* L.)			
phenolic extract from rockrose leaves and flowers (*Cistus incannus*)			
phenolic extract from pomegranate fruit (*Punica granatum*) L.			
phenolic extract from cranberry fruit (*Vaccinium macrocarpon*) + L-citrulline	548 mg of polyphenols and 2 g of L-citrulline per day	women between 18 and 75 years old	[84]
phenolic extract from grape seed (*Vitis vinifera*) + L-citrulline	548 mg of polyphenols and 2 g of L-citrulline per day	women between 18 and 75 years old	[84]
phenolic extract from barberry fruit (*Berberis vulgaris*)	10 g of dried barberry powder per day for 60 days	women and men around 55 years of age	[64]
phenolic extract from the fruit of colocynth watermelon (*Citrullus colocynthis*)	500 mg per kg of body weight per day	-	[46]

## Data Availability

No new data were created or analyzed in this study. Data sharing is not applicable to this article.
